# A Molecular and Chemical Perspective in Defining Melatonin Receptor Subtype Selectivity

**DOI:** 10.3390/ijms140918385

**Published:** 2013-09-06

**Authors:** King Hang Chan, Yung Hou Wong

**Affiliations:** Biotechnology Research Institute, State Key Laboratory of Molecular Neuroscience, Division of Life Science, The Hong Kong University of Science and Technology, Hong Kong; E-Mail: bskhchan@ust.hk

**Keywords:** melatonin, GPCR, MT_1_, MT_2_, subtype selectivity

## Abstract

Melatonin is primarily synthesized and secreted by the pineal gland during darkness in a normal diurnal cycle. In addition to its intrinsic antioxidant property, the neurohormone has renowned regulatory roles in the control of circadian rhythm and exerts its physiological actions primarily by interacting with the G protein-coupled MT_1_ and MT_2_ transmembrane receptors. The two melatonin receptor subtypes display identical ligand binding characteristics and mediate a myriad of signaling pathways, including adenylyl cyclase inhibition, phospholipase C stimulation and the regulation of other effector molecules. Both MT_1_ and MT_2_ receptors are widely expressed in the central nervous system as well as many peripheral tissues, but each receptor subtype can be linked to specific functional responses at the target tissue. Given the broad therapeutic implications of melatonin receptors in chronobiology, immunomodulation, endocrine regulation, reproductive functions and cancer development, drug discovery and development programs have been directed at identifying chemical molecules that bind to the two melatonin receptor subtypes. However, all of the melatoninergics in the market act on both subtypes of melatonin receptors without significant selectivity. To facilitate the design and development of novel therapeutic agents, it is necessary to understand the intrinsic differences between MT_1_ and MT_2_ that determine ligand binding, functional efficacy, and signaling specificity. This review summarizes our current knowledge in differentiating MT_1_ and MT_2_ receptors and their signaling capacities. The use of homology modeling in the mapping of the ligand-binding pocket will be described. Identification of conserved and distinct residues will be tremendously useful in the design of highly selective ligands.

## 1. Introduction

### 1.1. Melatonin and Melatonin Receptors

Melatonin (*N*-acetyl-5-methoxytryptamine) is a potent free radical scavenger and a regulator of redox-active enzymes in many plants [1], while in animals and humans, it is an important hormone with renowned regulatory roles in the mammalian circadian rhythm [2]. In both nocturnal and diurnal species, melatonin is produced by the pineal gland during the dark phase at night and its production is acutely suppressed by light. With the continuing output of the pineal gland, melatonin circulates in a physiologically active concentration at night [3] and induces the nocturnal melatonin signal that encodes time of day and length of day information to the central biological clock [4].

The primary role of melatonin in circadian phase readjustment, sleep initiation and support of sleep maintenance are largely mediated by the membrane receptors and to these effects, melatonin has been widely used in the treatment of chronobiological disorders such as seasonal affective disorders, insomnia and sleep disorders caused by blindness, shift work and jet lag [5–8]. A general formulation of 3 mg of melatonin is widely available as a nutritional supplement in the United States for producing pharmacological melatonin levels in the circulation [9]. Administration of 5 mg of melatonin before an imposed sleep period could advance the sleep phase in individuals with delayed sleep phase disorder without affecting total sleep duration [10,11]. Sustained melatonin treatment has also been shown to entrain rest-activity rhythms in blind people with free running disorder [12]. For travelers across multiple time zones, a daily regimen of melatonin of between 0.5 and 5 mg after arrival appears to be effective in reducing jet lag symptoms [13].

Melatonin also regulates an immense diversity of physiological and pathophysiological processes, including retinal physiology, seasonal reproductive cycles, cancer development and growth, immune modulation, antioxidation and free radical scavenging, mitochondrial respiration, cardiovascular function, bone metabolism, intermediary metabolism and gastrointestinal physiology [4,14,15]. Hence, melatonin was suggested to have a variety of clinical applications such as inhibiting the proliferation of various types of cancer cells [16] and exhibiting immunomodulatory properties [17]. As a potent antioxidant and free radical scavenger, melatonin offers neuroprotective and anti-inflammatory effects to neuronal cells from free radical and neurotoxin-induced damage and displays potential therapeutic benefits in treating neurodegenerative diseases including Parkinson’s disease, Alzheimer’s disease, muscular sclerosis, stroke and neuroendocrine disorders [18–22].

### 1.2. G Protein-Coupled Melatonin Receptors

Besides its free radical scavenging property, melatonin exerts most of its major physiological actions by interacting with seven-pass transmembrane G protein-coupled melatonin receptors. The first melatonin receptor was cloned from *Xenopus laevis* immortalized melanophore mRNAs which was expressed only in non-mammalian species [23]. Subsequently, cDNAs encoding two different forms of human melatonin receptors have been cloned and denoted as types 1 and 2 melatonin receptors (MT_1_ and MT_2_, respectively) [24,25]. The binding characteristics of the two receptor subtypes are similar, exhibiting subnanomolar affinities for melatonin [26]. MT_1_ and MT_2_ are separately mapped to chromosome 4q35.1 and 11q21-22, respectively, and encoding two proteins with 350 and 365 amino acids, respectively [24,27], sharing 55% overall identity and 70% within membrane domains [24,25]. Like many other G protein-coupled receptors (GPCRs), melatonin receptors are potentially glycosylated at their extracellular *N*-termini, and protein kinase C (PKC), casein kinase 1 and 2, and protein kinase A (PKA) phosphorylation sites are found in the intracellular *C*-terminal tails [26], which are involved in the functional regulations. An orphan GPCR, GPR50, shares 45% amino acid sequence identity with MT_1_ and MT_2_ [28], but its function is poorly understood. In fact, GPR50 does not bind melatonin at all. Intriguingly, co-expression studies showed that GPR50 dimerizes with either MT_1_ or MT_2_ and inhibits agonist binding of the MT_1_ but not MT_2_ and prevents the recruitment of intracellular signaling partners such as G proteins and β-arrestins to the receptor dimers [29]. Although non-validated, melatonin has been suggested to act as a ligand of the orphan nuclear receptor of the retinoic acid receptor family, named as retinoid Z receptor (RZR) and retinoid acid receptor-related orphan receptor (ROR) [30]. The RZR/ROR subfamily consists of three subtypes (α, β, γ) and four splice variants of the α–subtype, with RORα1 and RORα2 receptors being involved in immune modulation and are suggested to regulate cytokine production by immune cells upon melatonin binding [31]. Both MT_1_ and MT_2_ are distributed in neuronal and peripheral tissues [15,32]. MT_1_ and MT_2_ are found in the suprachiasmatic nucleus (SCN) at high densities [33] and their expression levels are synchronized with the diurnal rhythm [34]. In addition to the SCN, functional melatonin receptors are mainly localized in the brain [35] and many peripheral tissues, including the ovary [36], testis [37], arteries and heart [38], liver and kidney [39], adipocytes [40] and the immune system [26,31,41]; reviewed in references [26] and [32].

## 2. Melatonin Receptor Signaling

As a member of the superfamily of G protein-coupled receptors, melatonin receptors are capable of influencing a number of signaling cascades through the heterotrimeric guanine nucleotide binding proteins. Activation of melatonin receptors leads to the dissociation of the heterotrimeric G proteins, and the Gα subunit and Gβγ complex thereby interact with various downstream effectors. Upon receptor activation, melatonin receptors primarily inhibit adenylyl cyclases (AC) via the pertussis toxin (PTX)-sensitive G_i_ proteins (Gα_i2_ and Gα_i3_ isoforms) [42]. The decline in cAMP subsequently suppresses protein kinase A activity and nuclear factor CREB (cAMP responsive element binding protein) phosphorylation. Melatonin has been shown to play a role in the rhythmic regulation of clock gene expression via the AC/cAMP pathway [43]. Phosphorylated CREB can bind to the CRE site on the *mPER1* promoter and enhance the clock gene expression [44]. The melatonin-induced signaling cascade may modulate the circadian rhythm of the SCN through counteracting the effect of pituitary adenylyl cyclase activating peptide (PACAP) in the formation of phosphorylated CREB [45].

In addition to the cAMP-dependent signaling, G_i_-coupled melatonin receptors can utilize PTX-insensitive G_s_, G_z_ and G_16_ for signal propagation [46]. Through G_q_-coupling or the dissociation of βγ subunits of G_i_, melatonin stimulates the activity of phospholipase C (PLC), which in turn converts phosphatidylinositol (PIP_2_) into diacylglycerol (DAG) and inositol 1,4,5-triphosphatase (IP_3_). The elevated level of second messengers activate protein kinase C (PKC) and calcium signaling by calmodulin kinases (CaMK), thereby stimulating the mitogen-activated protein kinase (MAPK) cascade, including ERK, JNK and p38. The extracellular signal-regulated kinase (ERK) is mainly stimulated by growth factors, while c-Jun *N*-terminal kinase (JNK) and p38 MAPK are more responsive to cellular stress and cytokines [47]. JNK is capable of modulating the activities of several transcription factors including c-Jun, ATF-2 and Elk-1, to promote activating protein-1 (AP-1) transcriptional activity and to induce c-fos activation [48,49]. Thus, melatonin may regulate gene transcription via different pathways, through the activation of MAPK cascades, or through the suppression of CREB by inhibition of adenylyl cyclase. The role of melatonin in transcriptional events has been demonstrated in MCF-7 human breast cancer cells, which endogenously express the MT_1_ receptor. Treatment of MCF-7 cells with melatonin induces activation of JNK and ERK [46]. The observed phosphorylation of MAPK is postulated to upregulate the activity of c-fos, which dimerize with c-jun to promote AP-1 transcription activity in cell proliferation, differentiation, survival and apoptosis.

The presence of both MT_1_ receptor and G_16_ in hematopoietic cells suggests that the melatonin signaling plays an important role in hematopoietic development and immune regulation. In fact, melatonin can regulate cytokine production and has been reported to activate T helper cells through the induction of interleukin-2 (IL-2), as well as triggering interleukin-6 (IL-6) mediated monocyte activation [50]. The functional coupling between G_i_-coupled melatonin receptor with G_16_ has previously been shown to induce JNK phosphorylation *in vitro*, which allows melatonin receptors to modulate transcriptional events. In native Jurkat T cells, activation of MT_1_ receptor induces biphasic phosphorylation of signal transducer and activator of transcription 3 (STAT3) at Ser^727^ and Tyr^705^ through G_16_. STAT Ser^727^ phosphorylation occurred in the early phase, whereas STAT3 Tyr^705^ phosphorylation appeared only after prolonged exposure to 2-iodomelatonin [51]. The MT_1_/G_16_ mediated phosphorylation of STAT3 Ser^727^ is accompanied by elevated productions of IL-6, GM-CSF and CXCL-8, which serves as an autocrine signal to induce the late Tyr^705^ phosphorylation. The release of various cytokines also suggests a role for melatonin in promoting the differentiation of naive T cells into efficient B-lymphocyte helper T cells and the induction of neutrophil chemotaxis [52,53]. As STAT3 regulates gene transcription in cell proliferation and differentiation, it raises the possibility that melatonin may modulate the immunofunctions of hematopoietic cells and participate in different immunoenhancing events by mediating distinct Ser^727^ and Tyr^705^ phosphorylations.

Functionally, MT_1_ and MT_2_ have distinctive physiological roles. For example, MT_1_ mediates melatonin-regulated cardiac vasoconstriction [33,38,45], whereas MT_2_ activation dilates cardiac vessels and modulates inflammatory as well as immune responses [45,54,55]. The use of subtype-selective antagonists and transgenic animals has further demonstrated the different pathways mediated by the two receptor subtypes. MT_1_ knockout mice have improper sensorimotor gating and depression, indicating that the MT_1_ signaling is critical for normal brain function [56]. Another behavioral effect reported in knockout mice study is a deficit in memory based on the elevated plus-maze test in MT_2_ knockout mice [57]. However, the two receptors have been suggested to act in a complementary way in the SCN to modulate the body’s circadian rhythms as well as the sleep-wake cycle. Studies in mice showed that the neuronal firing in the SCN is suppressed by MT_1_ thereby implicating MT_1_ in sleep promotion [33]. A more recent study showed that MT_1_ may also modulate biological clock-related gene expressions as the expression of most clock genes is reduced in the pituitary of MT_1_ knockout mice but not in MT_2_ knockout mice [58]. On the other hand, it has been shown that the phase advancement of rat SCN neuron firing is mediated by MT_2_, hence implying its role in causing phase-shifting of the circadian rhythmic activity of SCN neurons [45,59]. These effects have been confirmed for MT_1_ on SCN slices by pharmacological experiments [33] or by *in vivo* treatment with the MT_2_ selective antagonist 4P-PDOT for MT_2_ [60]. While the melatonin receptor subtypes may work in concert to regulate various chronobiotic and homeostatic responses, the distinct roles of MT_1_ and MT_2_ spur interest in developing subtype-specific pharmacological agents to pinpoint their individual roles in the regulation of circadian rhythmicity, or promoting sleep without phase-shifting the circadian clock.

## 3. Development of Synthetic Melatoninergics

**Figure 1:**
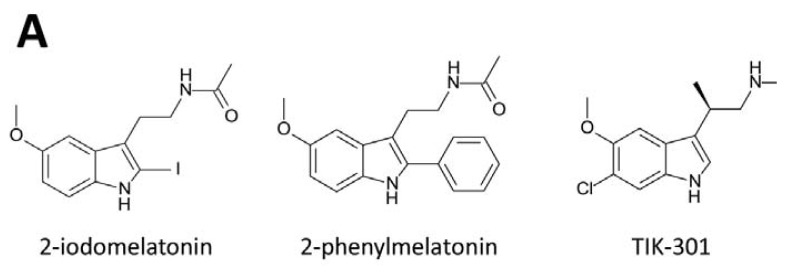

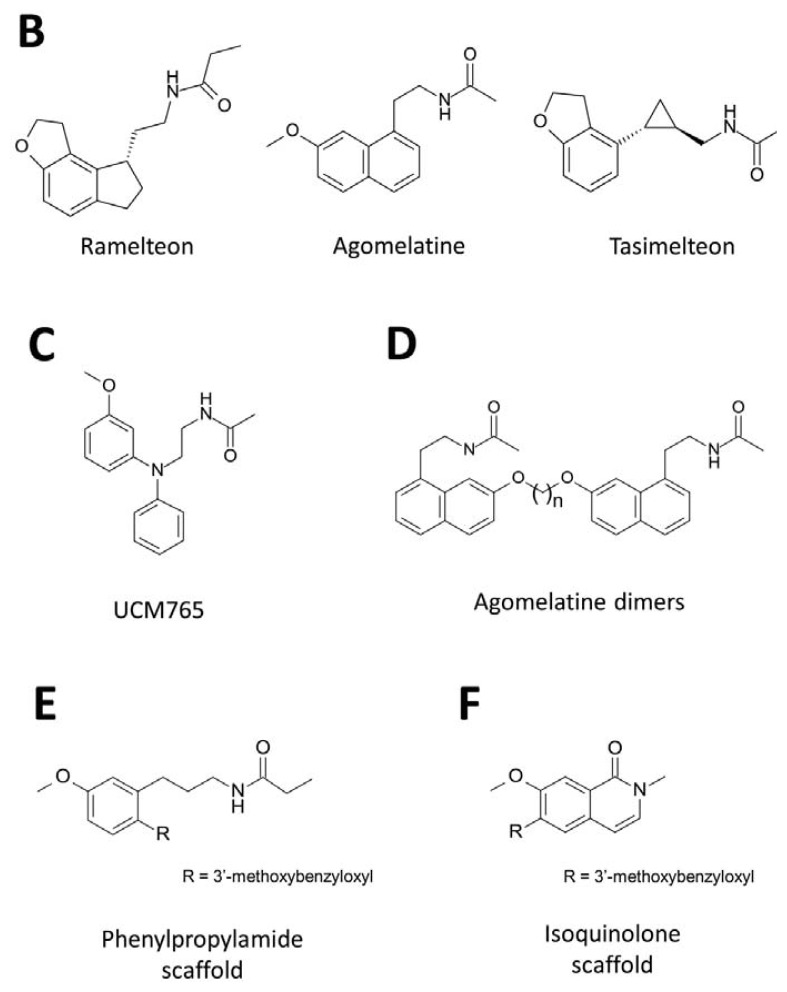
Structures of melatonin ligands. (**A**) 2-iodomelatonin, 2-phenylmelatonin and TIK-301; (**B**) Ramelateon, Agomelatine and Tasimelteon; (**C**) MT_2_ selective ligands, UCM765; (**D**) MT_1_ selective ligands, agomelatine dimmers; (**E**) Substituted *N*-[3-(3-methoxyphenyl) propyl] amide (*N*-(3-{5-Methoxy-2-[2-(3-methoxy-phenyl)-methyleneoxy]-phenyl}-propyl)-propionamide); (**F**) Substituted isoquinolone (7-Methoxy-6-(3-methoxy-benzyloxy)-2-methylisoquinolin- 1(2*H*)-one).

The therapeutic potential of melatonin is limited by its non-specific actions at multiple receptors as well as its unfavorable pharmacokinetic properties, such as high first-pass metabolism, short half-life and poor oral bioavailability [61]. Hence, much work has been undertaken to discover and develop new classes of melatoninergic ligands with improved pharmacological properties including receptor subtype selectivity and higher binding affinity. Melatoninergics agonists such as 2-iodomelatonin, 2-phenylmelatonin [62,63] and TIK-301 (β-methyl-6-chloromelatonin) [64] were therefore developed (Figure 1A), resembling the indolic core structure of melatonin with slightly higher affinity toward MT_2_. Bioisosteric replacements of the melatonin structure have also been widely adopted resulting in numerous non-indoleamine melatoninergics with heterocyclic scaffolds appearing in the literature. The ever-growing list includes indane, naphthalene, benzoxazole and benzofuran, fluroene, carbazole, quinoline, isoquinoline, benzopyran, benzothiazole, benzoxazine, benzothiazine, benzoxadole, azaindole, benzylpiperidine, biphenyl, pyridine, aryloxyanilide and phenylpropylamide [65–68]. Ramelteon (Figure 1B) is an indane derivative with critical features for melatonin receptor binding and shows high binding affinity at both MT_1_ and MT_2_ receptors (*K*_i_ CHO-hMT_1_ = 14 pM; *K*_i_ CHO-hMT_2_ = 45 pM) [61,69]. Another widely used strategy to obtain potent melatoninergic ligands is by replacement of the indole moiety with aromatic fragments. Agomelatine (Figure 1B) is a naphthalene derivative [70,71] that retains a similar binding affinity as melatonin but possesses both melatonin agonist and serotonin antagonist properties (*K*_i_ CHO-hMT_1_ = 0.1 nM; *K*_i_ CHO-hMT_2_ = 0.12 nM; *K*_i_ CHO-h5-HT_2C_ = 708 nM) [72,73]. By mimicking the indane structure of Ramelteon, a dihydrobenzofuran scaffold is linked to the ethylamidomethyl chain by a cyclopropyl ring. Tasimelteon (Figure 1B) displays melatoninergic activity with minimal structural requirements for receptor binding [74]. A diverse array of synthetic melatoninergic ligand from different structural scaffolds has been developed and their receptor affinity, intrinsic activity, subtype selectivity and metabolism were studied in relation to their chemical design. Despite these advances, very few subtype-selective melatoninergic ligands are available for research purposes, let alone drug development. Luzindole and 5-methoxyluzindole are among the early identified melatoninergics showing modest MT_2_ selectivity. The incorporation of 2-benzyl group appears to reduce binding affinity at the MT_1_, leading to MT_2_ selectivity [75]. Similarly, analogs like N0889 and DH97 are endowed with a higher affinity for MT_2_ receptor and *K*_i_(MT_1_)/*K*_i_(MT_2_) ratios ranging from 31- to 89-fold [75,76]. Further variations of the 2-benzyl group have been made by cyclization to constrain its orientation, yielding tetracyclic compounds like K185 and IIK7, which also exhibited 90- and 132-fold higher affinity for the MT_2_ than for the MT_1_ receptor, respectively [77]. The role of an aromatic group right next to the alkylamide chain in subtype selectivity is further supported by other melatoninergics with non-indole scaffolds. Binding affinity of 4P-PDOT, S24773, S24014 and S28407 display over 100-fold preferences toward the MT_2_ receptor [72,78,79]. Despite being receptor subtype selective, none of these melatoninergic agents had been clinically developed and marketed as a treatment for circadian rhythm related sleep disorders. The development of a subtype-selective drug candidate may result in a safer medicine with more favorable pharmacological profiles. UCM765 (Figure 1C) is a relatively simple *N*-phenylaniline-based compound which preferentially binds to MT_2_ with an affinity two orders of magnitude higher than to MT_1_ [80,81]. A metabolically more stable analog, UCM924, has also been developed without compromising binding affinity. Their MT_2_ selectivity are believed to be more favorable for re-entrainment of the circadian rhythm and alleviation of symptoms related to shifted or poorly coupled circadian oscillations. Discovery of MT_1_-selective compounds is unexpectedly rare. A bulky substituent replacing the 5-methoxy group of melatonin or its naphthalenic analog gives rise to a series of modestly MT_1_-selective compounds. A key feature is that most of them are dimeric derivatives resembling two molecules of agomelatine attached to both ends of apolymethylene chain (Figure 1D) [79]. The length of the linking chain can vary from two to eight, but three gives the highest selectivity ratio. Functional characterization of one of these dimeric melatoninergics reveals its antagonistic activity with approximately 40-fold MT_1_ selectivity. Another series of MT_1_-selective ligands are *N-*(anilinoalkyl)amides bearing 3-arylalkyloxy or 3-alkyloxy substituents at the aniline ring. Derivative with a phenylbutyloxy substituent was shown to be a potent partial agonist, displaying 78-fold selectivity for the MT_1_ receptor [82].

The emergence of melatoninergic agonist, partial agonist and antagonist not only helps to delineate melatonin receptor mediated actions, but many of the compounds were also used as pharmacological tools in rationalizing their structure activity relationships (SAR), providing new insights into the development of new classes of melatoninergic therapeutic agents. Previous studies of the structure-activity relationship have revealed that the 3-acylaminoethyl chain and 5-methoxy groups on the indole ring of melatonin are the crucial components in receptor binding; removal of either the acylaminoethyl chain or the methoxy group would lead to significant loss of receptor affinity [62,83]. The size of the amide terminal group on the acylaminoethyl chain appears to modulate ligand affinity on the melatonin receptors. Extending the acetyl group to propionyl endowed the compound with improved binding affinity and potency, but substituents higher than butyl or composed of branched structure have an adverse effect on the affinity. There have been reports that substitutions with cyclopropylcarbonyl or cyclobutylcarbonyl group would shift the intrinsic activity from agonist to antagonist [84,85]. The relative distance of the amide group and the methoxyl group is also critical for ligand-receptor interaction. Melatonin analogs with the methoxyl group moving to positions 4, 6 or 7 exhibited reduced affinity, indicating that 5-methoxyl group is optimal for receptor binding [85,86]. Replacement of the 5-methoxyl group by hydrogen, hydroxyl or alkoxy groups also decreased affinity, while halogen substituents retained both affinity and intrinsic activity [87]. Affinity of melatonin can be further enhanced by substituting a halogen, methyl or a phenyl group at the C2 position of the indole ring. A series of substituted analogs also displays higher affinity for the MT_2_ receptor, indicating that these substituents are better tolerated in the MT_2_ binding pocket and are likely interacting with an auxiliary binding subdomain in the receptor [88,89]. Furthermore, it has been subsequently revealed that the indole scaffold does not actually play a major role in the receptor binding. Either shifting the position of the nitrogen atom in the pyrrolidine ring or replacing indole with other aromatic fragments does not generally affect the receptor affinity [80,90]. With the appropriately positioned acylaminoethyl and methoxy groups, bioisosteres, such as Ramelteon and agomelatin, have demonstrated potent melatoninergic activity similar to the indole-based derivatives [85,89].

A number of synthetic melatoninergics with improved pharmacokinetic properties compared to melatonin have been developed into clinical uses, but none of them exhibits selectivity towards either receptor subtype [91]. The development of a subtype-selective drug candidate may result in a safer medicine with more favorable pharmacological profiles. A novel series of substituted *N*-[3-(3-methoxyphenyl)propyl]amides were identified in our laboratory displaying superb binding affinities toward human MT_2_, and MT_1_ to a lesser extent [92,93]. Similar to melatonin, a terminal simple alkyl group (up to propyl) attached on the propylamide chain progressively improves the intrinsic activity, whereas a terminal phenyl substitute is devoid of any binding. Particularly, introduction of a 3′-methoxyl group to the benzyloxyl substitute incorporated at C6 position of the phenylpropylamide scaffold (analogous to C2 position of melatonin) dramatically enhances the binding affinity toward MT_2_ but not MT_1_ (Figure 1E). Furthermore, unpublished results in our laboratory indicate that a 3′-methoxybenzyloxyl group linked with a bicyclic isoquinolone scaffold (Figure 1F) without the protruding alkylamide chain can still bind MT_2_ at modest to high affinity when compared with melatonin, and these compounds are basically inactive toward MT_1_. Substitution at the 6 position provided a potent agonist, which was endowed with high MT_2_ binding affinity (*K*_i_ = 26.6 nM) and 139-fold selectivity for the MT_2_ receptor. The physiological significance of such exclusively MT_2_-selective compounds awaits further studies.

## 4. Homology Models of Melatonin Receptors

**Figure 2:**
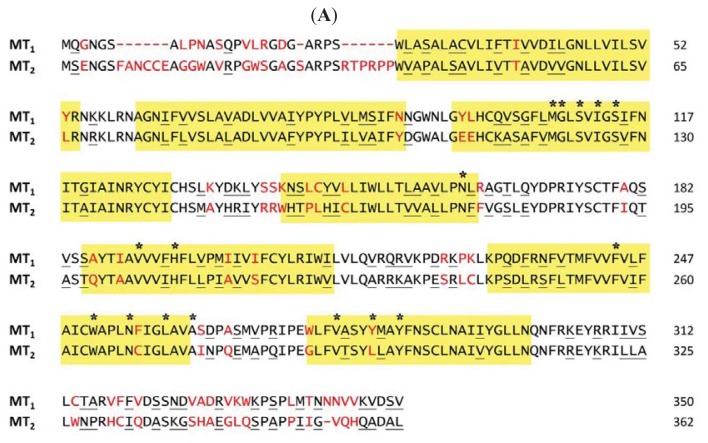

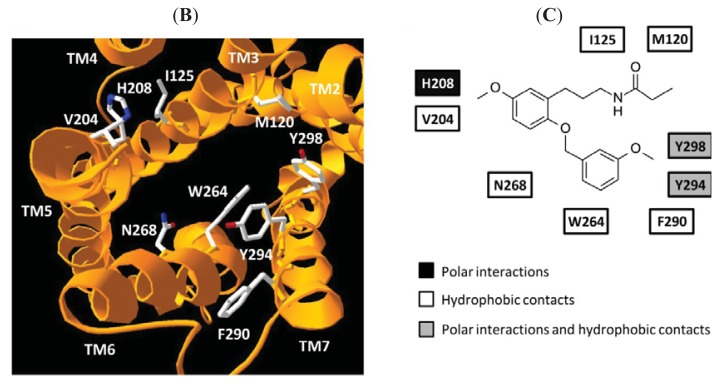
Ligand binding pocket of phenylpropylamide-bound MT_2_ receptor structure. (**A**) Superposition of human MT_1_ and MT_2_ receptor sequences. Residues corresponding to the transmembrane regions are overtyped in yellow. Identical or conserved residues are in black, conserved substitutions are underlined. Residues differing from one subtype to the other are in red. An asterisk (*) indicates a previously characterized single point mutation to the hMT_2_ melatonin receptor; (**B**) Extracellular view of the model of the MT_2_ receptor. Only the proposed amino acid residues participated in the binding of phenylpropylamide (Met120, Ile125, Val204, His208, Trp264, Asn268, Phe290, Tyr294 and Tyr298) are shown. The model of the MT_2_ receptor was created by using homology modeling from the published crystal structure of the human β2 adrenergic receptor solved at 2.4 Å resolution (PDB access code 2RH1) [111]. Figure was generated using DeepView/Swiss-PdbViewer v3.7; (**C**) Schematic representation of the interactions between MT_2_ receptor and the ligand phenylpropylamide. Mutation of amino acid in black box indicates potential polar interactions. Mutations of amino acids in white boxes indicate potential hydrophobic interactions, and grey boxes indicate both potential polar interactions and hydrophobic interactions.

Similar to other rhodopsin-like GPCRs, melatonin receptors contain seven putative transmembrane helices connected by three intracellular and three extracellular loops. These helices are believed to superpose with that of rhodopsin-like receptors and have common residues at a conserved position. The structural similarity has allowed the interactions between melatonin receptors and their ligands to be predicted through a combination of site directed mutagenesis and homology modeling approaches. Sequence alignment of MT_1_ and MT_2_ showed that the position of a conserved histidine in TM5 (His195 for MT_1_ and His208 for MT_2_) is identical to that utilized in the ligand binding site of many other GPCRs, conferring an important role of the residue in ligand recognition (Figure 2A). Substitution of the histidine residue with alanine reduced the binding affinity of melatonin but did not affect the affinity for the antagonist luzindole [94,95]. Previous studies of structure-activity relationship have revealed that the 5-methoxy group of melatonin or equivalent methoxy group of other melatoninergic agonists is a crucial component in receptor binding [87]. The oxygen of the methoxy group has been postulated to form a hydrogen bond with the histidine residue on TM5, which is essential for receptor activation.

Residues located within TM3, 5, 6 and 7 have also been extensively studied by site-directed mutagenesis, but it appeared that the conserved histidine is the only common residue correlated to the binding site of the two subtypes. TM3 of rhodopsin-like GPCRs contains a relatively large number of cysteine, serine and threonine residues than the other TM domains. These residues can form hydrogen bonds with the peptide backbone and facilitate receptor conformational change at different functional states. Ser110 and Ser114 in TM3 of MT_1_ seem to be involved in agonist binding through interacting with the acetamidoethyl chain of melatonin [96]. Mutations of serine residues to alanine reduced melatonin affinity, while the binding of luzindole was unaffected, inferring the subset of residues for binding agonist and antagonist may differ. In contrast, the equivalent TM3 serine residues (Ser123 and Ser127) on MT_2_ are not important for ligand binding [94]. The distinct tolerance to conserved serine mutations hinted of structural divergence between the two subtypes.

Ligand binding interaction was relatively less evident in MT_2_ receptor and different hypotheses have been proposed to predict the putative binding site. Gerdin *et al.* suggested that both the asparagine in the TM4 (Asn175) and the conserved His208 participate in the interaction with the 5-methoxy group of melatonin, while two residues in the TM6 domains (Phe257 and Trp264) facilitate the binding of aromatic compounds such as luzindole and 4P-PDOT through π-π interaction [94]. On the other hand, Mazna and colleagues have identified several residues in TM3 (Met120, Gly121 and Ile125), TM5 (Val204), TM6 (Leu272 and Ala275) and TM7 (Val291 and Leu295) that are crucial for ligand binding as mutations at these positions reduced the binding affinity for melatonin. The subset of residues identified was found to be involved in agonist binding possibly via hydrophobic interactions with the indole group and *N*-acetyl group of 2-iodomelatonin [97,98]. The authors also hypothesized a potential role for Asn268 and Tyr298 in facilitating the specific interaction of His208 with the 5-methoxy substituent of melatonin.

Based on the hypothesis proposed by Gerdin *et al.* [94], Farce *et al.* re-orientated the helix bundles in their proposed receptor models to represent an active form of melatonin receptor upon binding melatonin [99]. To place the critical residues (Ser110, Ser114 and His195 in MT_1_; Asn175 and His208 in MT_2_, respectively) in proper position to bind melatonin, the authors have to rotate TM3 and TM5 helices of MT_1_ receptor in a clockwise manner, while rotating TM4 of MT_2_ counter-clockwise. The distinct conformations adopted by the two receptor subtypes in response to endogenous melatonin binding may confer functional selectivity in signaling upon receptor activation.

Mor *et al.* have published SAR data showing that the incorporation of a distant 2-benzyl substituent on the indole scaffold of melatonin specifically enhances MT_2_ binding [100]. The data gathered suggest that an additional binding cavity formed by a large group of hydrophobic amino acid side chains may exist in the MT_2_ receptor binding site to accommodate the aromatic out-of-plane substituent. To identify the putative hydrophobic pocket, homology models of MT_1_ and MT_2_ receptors have been built using the crystal structure of bovine rhodopsin as a template. In the MT_2_ model proposed by Rivara *et al.*, the aromatic ring of the reference MT_2_ selective antagonist *N*-[1-(4-chloro-benzyl)-4-methoxy-1H-indol-2-ylmethyl]-propionamide lied in a putative hydrophobic pocket located between TM3, TM5 and TM6, and in close vicinity to the side chain of Trp264 upon ligand docking [101]. Molecular dynamics simulations confirmed that the distance between Trp264 and the aromatic substituent of the antagonist is observed to be closer in MT_2_ than that of the MT_1_ receptor complex. The two interacting groups form a stable π-π interaction when in close proximity and enhance MT_2_ selectivity. The observed interaction, however, limits the conformational freedom of the CWXP motif in TM6 and prevents receptor activation, thus providing an explanation for the antagonist activity.

Among the family of rhodopsin-like GPCRs, melatonin receptors and opsins are phylogentically close and may share a common ancestor. Despite their sequence similarity, the partially active opsin structure is suggested to be conformationally different from a ligand binding activated receptor, inferring diverse mechanisms underlying GPCR activation throughout the family. The homology modeling of small molecule binding aminergic receptor therefore may provide a reference set of amino acids for determining the ligand binding residues in the melatonin receptor [102,103]. Protein sequence alignment of a diverse array of vertebrate receptor orthologues revealed that amino residue 6.48 (equivalent to Trp264 in MT_2_) is conserved in most rhodopsin-like GPCRs and is likely to play a general role in receptor activation by stabilizing conformational alternation of side chains upon agonist binding [102,104]. Base on the aminergic binding site defined by evolutionary trace analysis, Gloriam *et al.* predicted 12 positions on TM3 (3.32, 3.33, 3.37 and 3.40), TM5 (5.42, 5.43, 5.46 and 5.47), TM6 (6.51 and 6.52) and TM7 (7.42 and 7.43) are involved in the generic binding site (corresponding residues in MT_1_ and MT_2_ receptors are listed in Tables 1 and 2) [105]. The subset of residues identified is in agreement with the MT_2_ receptor homology model proposed by Mazna and colleagues [97,98].

Agomelatine is an antidepressant which binds specifically to melatonin and type 2C serotonin (5-HT_2C_) receptors [70,71]. This cross reactivity suggests that both receptors may share a similar structure-activity relationships profile upon binding agomelatine. The modeling of the agomelatine-bound state of the serotonin receptor therefore may allow us to identify important structural features that are may be similarly present in the melatonin receptors. An extensive screening of antagonist-bound 5-HT_2C_ models suggested that antagonists are caged between TM3, TM5, TM6 and TM7, and agomelatine adopts a conformation wherein its protonated amine group interacts with Asp134 (3.32) and Tyr358 (7.43) through hydrogen bonding [107]. As observed in the respective crystal structures, the naphthalene ring of agomelatine also participates in π-π stacking binding interactions with the hydrophobic cluster comprising Trp324 (6.48), Phe327 (6.51) and Phe328 (6.52) [108]. These two identified structural features are not only valuable to the design of new antidepressants, they also help to further our understanding of the nature of residues that are required for receptor-ligand interaction.

Homology modeling approaches have identified a subset of residues and potential structural determinants that appear to be critical for receptor ligand binding [82,109]. However, the reliability of the prediction is susceptible to the degree of similarity between the target sequence and the template of crystallized receptors. Comparative analyses of prospective modeled ligand-receptor complexes generated from distinct classes of GPCR templates suggest that 35%–40% sequence identity boundaries are required for reliable homology modeling in general [110]. Although modeling between closely related receptor subtypes, and especially, among the same class of GPCRs is generally successful, distant homology modeling based on a phylogenetically remote receptor template often fails to produce accurate receptor models due to significant deviation in structures [109]. Melatonin shares a low sequence identity with the available crystallized receptor, and hence, receptor modeling is usually accompanied by experimental data from mutagenesis studies. Recent resolution of crystal structures of aminergic receptors allows melatonin receptor homology models to be built by using a structurally similar receptor template and this approach may facilitate our understanding of the melatonin receptor structure.

On the basis of mutagenesis studies demonstrating that the conserved histidine residue (His195 for MT_1_ and His208 for MT_2_) is important for melatonin binding and activation [94,95], possible interaction of His208 in MT_2_ with the 3-methoxy group of phenylpropylamide was probed by assessing the potency difference between the wild-type and mutant receptors. When the histidine residue in the MT_2_ receptor was mutated to alanine (His208Ala), MT_2_-selective phenylpropylamide compounds exhibited a significant loss in potency and efficacy (unpublished data). Despite their difference in subtype selectivity, the data suggests that the interaction between the methoxy group and the histidine is critical for both melatonin and phenylpropylamide binding, and the two agonists may stabilize the receptor binding pocket conformation in a similar manner. In the presence of the selectivity determinant 3′-methoxybenzyloxyl group at C6 position of the phenylpropylamide scaffold, compounds preferentially activate MT_2_ over MT_1_. According to receptor models proposed by Mor *et al.* [100] and Rivara *et al.* [101], an additional binding cavity present in MT_2_ is able to accommodate the aromatic substituent, thereby increasing the MT_2_ affinity of phenylpropylamide compounds bearing a 3′-methoxybenzyloxyl moiety. Base on the homology modeling of melatonin and serotonin receptors, such a cavity is most likely formed by a large group of hydrophobic amino acid side chains located at TM6 (Trp264, Asn268) and TM7 (Phe290, Tyr294, Tyr298) (Figure 2B,C). Molecular interactions between the 3′-methoxyl substituent on the benzyl ring and the MT_2_ receptor, however, are less understood. To characterize structural features responsible for subtype selective ligand binding at the MT_2_ receptor, additional mutagenesis studies will need to include selective residues that participate in the formation of the putative hydrophobic binding pocket.

Lastly, a novel series of MT_2_-selective isoquinolone derivatives (Figure 1F) can bind to and activate the His208Ala MT_2_ receptor mutant. These compounds possess a substituted 3-methoxylbenzyloxyl substituent at their bicyclic scaffold but have little structural resemblance to melatonin. They exhibit an EC_50_ comparable to that of melatonin towards MT_2_ and are totally inactive at MT_1_. Functional assays also demonstrated that the compound-induced response in His208Ala was essentially indistinguishable from that observed with the wild-type receptor, leading to the speculation that agonists can bind to the MT_2_ receptor in at least two configurations as His208 may not be required for receptor activation. Identification of the residues of MT_2_ receptor that participate in MT_2_-selective ligand docking awaits further mutagenesis studies

## 5. Conclusions

Melatonin exerts its physiological actions through mediating multiple signaling pathways via transmembrane melatonin receptors. Parallel signaling mechanisms of melatonin also promote transcriptional activities by recruiting various kinases and transcription factors, acting on the downstream of signaling pathways in a cell type specific manner. Hence, melatonin displays a pleiotropic functional profile in addition to its primary role in regulating circadian rhythm and sleep promotion. With regard to the distinct role of melatonin receptor subtypes in chronobiology and immunomodulation, subtype-selective melatoninergics are of emerging interest since they may result in a safer medicine with more promising therapeutic effects. Further clarifications of receptor ligand molecular interactions will guide the development of melatonin receptor subtype-specific therapeutic agents while structural data remains relatively limited.

## Figures and Tables

**Table 1:** Characterizations of single point mutations to the hMT_1_ melatonin receptor.

Mutation	General TM numbering	Binding/functional characterizatics
S110A	3.35	*K*_d_ increased by 8-fold, *B*_max_ reduced by 10-fold. *K*_i_ increased by 9-fold. No change in K_i_ of luzindole [96].
S114A	3.39	*K*_d_ increased by 9-fold, *B*_max_ reduced by 4-fold. *K*_i_ increased by 4-fold. No change in *K*_i_ of luzindole [96].
H195A	5.46	EC_50_ of melatonin or 2-iodomelatonin reduced by 3–6 fold in yeast CPRG assay [95].

The equilibrium dissociation constant (*K*_d_) and the maximum binding capacity (*B*_max_) were determined in saturation studies using 2-[^125^I]-iodomelatonin. Unless otherwise specified, *K*_i_ represents the equilibrium dissociation constant of melatonin. Please refer to reference [106] for more details on mutagenesis studies of melatonin receptors.

**Table 2:** Characterizations of single point mutations to the hMT_2_ melatonin receptor

Mutation	General TM numbering	Binding/functional characterizatics
M120A	3.32	No change in *K*_d_, *B*_max_ reduced by 3-fold. *K*_i_ increased by 2-fold. *K*_i_ of 4P-PDOT reduced by 6-fold [98].
G121A	3.33	No change in *K*_d_ or *B*_max_. *K*_i_ increased by 3-fold [98].
G121I	3.33	No change in *K*_d_ or *B*_max_. *K*_i_ increased by 2-fold [98].
S123A	3.35	No change in *K*_d_ or K_i_. *B*_max_ reduced by 5-fold [94].
S127A	3.39	No change in *K*_d_ or *K*_i_. *B*_max_ reduced by 3-fold [94].
I125A	3.37	No change in *K*_d_ or *B*_max_. No change in *K*_i_ [98].
N175A	4.60	No change in *K*_d_ or *B*_max_. *K*_i_ increased by 4-fold [94].
V204A	5.42	NSB [97]
H208A	5.46	*K*_d_ increased by 2-fold, *K*_i_ increased by 4-fold. No change in *B*_max_ [94].
F257A	6.41	No change in *K*_d_ or *B*_max_. No change in *K*_i_ [94].
W264A	6.48	K_d_ reduced by 2-fold, no change in *K*_i_. *B*_max_ reduced by 23-fold [94].
N268A	6.52	NSB [98]
N268D	6.52	NSB [98]
N268L	6.52	NSB [98]
N268Q	6.52	No change in *K*_d_ or *B*_max_. No change in *K*_i_. [98]
L272A	6.56	NSB [97]
A275I	6.59	NSB [98]
A275V	6.59	No change in *K*_d_ or *B*_max_. No change in *K*_i_. [98]
V291A	7.36	NSB [98]
V291I	7.36	NSB [98]
L295A	7.40	NSB [98]
L295I	7.40	NSB [98]
L295V	7.40	NSB [98]
Y298A	7.43	NSB [97]

*NSB*, no specific binding. The equilibrium dissociation constant (*K*_d_) and the maximum binding capacity (*B*_max_) were determined in saturation studies using 2-[^125^I]-iodomelatonin. Unless otherwise specified, *K*_i_ represents the equilibrium dissociation constant of melatonin. Please refer to reference [106] for more details on mutagenesis studies of melatonin receptors.
